# Use of Natural Products as Chemical Library for Drug Discovery and Network Pharmacology

**DOI:** 10.1371/journal.pone.0062839

**Published:** 2013-04-25

**Authors:** Jiangyong Gu, Yuanshen Gui, Lirong Chen, Gu Yuan, Hui-Zhe Lu, Xiaojie Xu

**Affiliations:** 1 Beijing National Laboratory for Molecular Sciences, State Key Lab of Rare Earth Material Chemistry and Applications, College of Chemistry and Molecular Engineering, Peking University, Beijing, P. R. China; 2 Institute of Science and Technology, China Agricultural University, Beijing, P. R. China; Royal College of Surgeons, Ireland

## Abstract

**Background:**

Natural products have been an important source of lead compounds for drug discovery. How to find and evaluate bioactive natural products is critical to the achievement of drug/lead discovery from natural products.

**Methodology:**

We collected 19,7201 natural products structures, reported biological activities and virtual screening results. Principal component analysis was employed to explore the chemical space, and we found that there was a large portion of overlap between natural products and FDA-approved drugs in the chemical space, which indicated that natural products had large quantity of potential lead compounds. We also explored the network properties of natural product-target networks and found that polypharmacology was greatly enriched to those compounds with large degree and high betweenness centrality. In order to make up for a lack of experimental data, high throughput virtual screening was employed. All natural products were docked to 332 target proteins of FDA-approved drugs. The most potential natural products for drug discovery and their indications were predicted based on a docking score-weighted prediction model.

**Conclusions:**

Analysis of molecular descriptors, distribution in chemical space and biological activities of natural products was conducted in this article. Natural products have vast chemical diversity, good drug-like properties and can interact with multiple cellular target proteins.

## Introduction

Natural products (NPs) play an important role in drug discovery [1]–[3]. About more than 50 percent of FDA-approved drugs were NPs or natural products derivatives [4], [5]. Moreover, NPs have special selectivity to cellular targets [6]. Biologically active natural products would provide selective ligands for disease-related targets [7], and influence the disease-related pathways and eventually shift the biological network from disease status to the healthy status.

With the development of large-scale network analysis, researchers have recently begun to explore the action mechanism of bioactive compounds in the context of biological networks, e.g. drug-target network (DTN) [8]–[10], protein-protein interaction network [11], metabolic network [12], [13] and disease pathway [14]. However, most studies focused on few molecules. NPs possesses vast chemical diversity and so have enormous potential to find various different kinds of bioactive molecules [15]. Researchers have done statistics and analysis for natural products in several aspects, such as chemical diversity [15]–[18], property distribution [19], molecular scaffold [20]–[22], chemical space [23], [24] and comparison between NPs and other compound collections [22], [25], [26]. However, researchers seldom did comprehensive statistics on natural products and comparison between NPs and other types of compounds because it was difficult to obtain large quantity of data collection (both structures and annotations).

During the past decades, our laboratory has been focusing on pharmaceutically relevant natural products. In 2002, we established a 3D structure database of components from Chinese traditional medicinal herbs (CHDD) [27]. Right now, we constructed the Universal Natural Products Database (UNPD) to facilitate the high throughput virtual screening from natural products and the database comprised 197201 natural products now. To the best of our knowledge, UNPD is the largest non-commercial and freely available database for natural products (http://pkuxxj.pku.edu.cn/UNPD). UNPD comprised 197201 natural products from plants, animals and microorganisms. Based on the calculated molecular properties, we compared NPs and FDA-approved drugs in many aspects. We also explored the potential of use NPs as chemical library for drug discovery and network pharmacology by using both experimental and computational results.

## Methods

### 1. Collection of Natural Products and Approved Drugs

The natural products were collected from Reaxys, Chinese Natural Product Database (CNPD) [28], Traditional Chinese Medicines Database (TCMD) [29] and our CHDD [27]. The number of compounds and number of duplicate structures in each databases were listed **Table 1**. The 3D structures were generated by Discovery Studio. We use the absolute configuration of each natural product. For those ambiguous structures (e.g. R/S or Z/E is not clear), we create two absolute configuration and assign different number to each configuration. When one structure had two part (e.g. salts or adducts), the larger part was retained and the smaller part was deleted. The duplicates were removed according to InChIKey generated by Open Babel [30]. Therefore, each molecule in UNPD has unambiguous stereoconfiguration. All chemical structure were minimized in MMFF94 force field. The structure of approved drugs were downloaded from DrugBank.

**Table 1:** The number of compounds and number of duplicate structures in each databases.

Databases	CHDD	CNPD	TCMD	Reaxys
Total compounds	30564	57346	23303	171504
Used in UNPD	29759	41729	7528	118185
Duplicates	785	15617	15775	53319

### 2. Calculation and Statistics of Molecular Descriptors of NPs and Drugs

**Figure 1:**
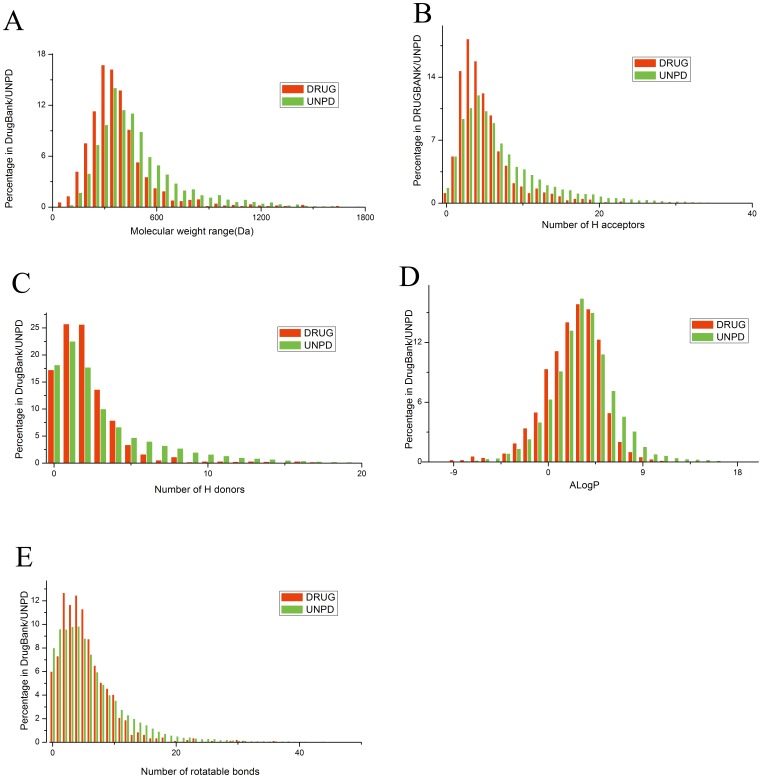
Distribution of five molecular descriptors of natural products and approved drugs.

Molecular descriptors of NPs and drugs in **Figure 1** and **Table 2** were calculated in Discovery Studio by using default parameters. PaDEL-Descriptor [31], a free software developed by National University of Singapore, was employed to calculate substructure-related molecular descriptor and 307 substructure descriptors.

**Table 2:** Statistics of molecular descriptors of natural products in UNPD and FDA-approved drugs in DrugBank.

Descriptors		Natural products in UNPD				Approved drugs		
	Mean	Median	Min	Max	Mean	Median	Min	Max
AlogP	2.788±3.352	2.710	−36.007	53.473	1.899±2.814	2.164	−12.834	14.242
Molecular_Weight	472.6±265.7	406.5	16.0	3973.5	360.8±199.1	322.1445	6.9	1639.9
Num_Rotatable_Bonds	6.6±6.8	5	0	140	5.5±4.9	4	0	44
Num_Rings	3.7±2.4	3	0	32	2.7±1.6	3	0	10
Num_Aromatic_Rings	0.9±1.3	0	0	20	1.3±1.1	1	0	8
Num_H_Acceptors	7.5±6.7	6	0	104	5.2±4.2	4	0	51
Num_H_Donors	3.4±4.1	2	0	64	2.3±2.6	2	0	23
Molecular_Volume	323.8±172.5	278.5	19.2	2576.2	238.0±128.1	217.8	6.8	1053.3
Molecular_Surface_Area	462.4±249.4	400.2	33	4020.9	347.7±186.4	312.45	16.6	1586.9
Molecular_Polar_Surface_Area	122.2±110.6	87.0	0	1917.9	93.9±84.1	75.0	0	878.8
Molecular_Fractional_PolarSurfaceArea	0.248±0.128	0.233	0	1	0.277±0.171	0.242	0	1
Molecular_SASA	683.0±295.7	612.5	0	5106.7	563.7±232.6	523.3	137.9	2167.1
Molecular_PolarSASA	194.9±169.3	142.1	0	3101.9	152.1±131.0	126.0	0	1321.8
Molecular_FractionalPolarSASA	0.268±0.144	0.242	0	0.967	0.272±0.171	0.239	0	0.933
Molecular_SAVol	591.7±253.7	531.5	0	4468.8	495.9±202.2	462.8	124.3	1900.9

Note: the descriptors of 197201 natural products in UNPD and 1380 FDA-approved small molecule drugs in DrugBank were calculated by Discovery Studio.

### 3. Chemical Space Analysis

**Figure 2:**
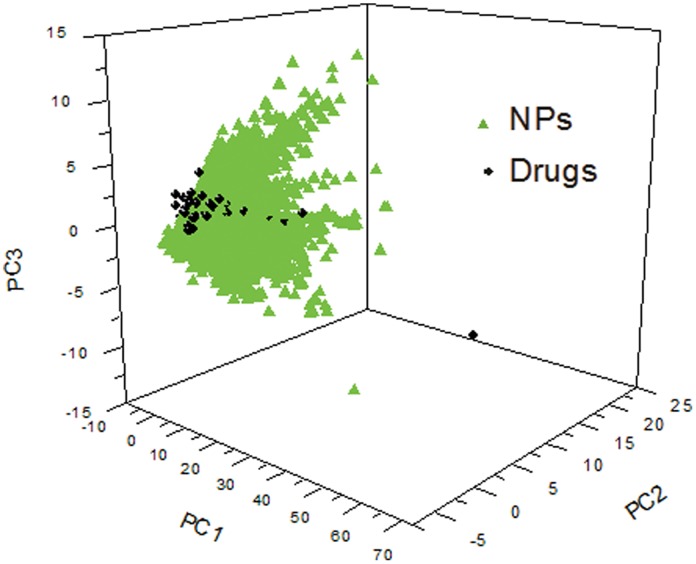
The distribution in chemical space according to principal component analysis of natural products in UNPD and FDA-approved drugs. The green triangles and black dots represent natural products and FDA-approved drugs, respectively.

Principal component analysis (PCA) was conducted in library analysis module of Discovery Studio and the input parameters were listed in **Table 2**. PCA was an orthogonal linear transformation technique which can transform the data into a new coordinate system, which is in three-dimensional system in our analysis. The variance of the data which was maximized on the first coordinate was called first principal component. The rest of variance maximized on the second coordinate, and so on. The PCA model was built with 8 descriptors: AlogP, Molecular_Weight, Num_H_Donors, Num_H_Acceptors, Num_RotatableBonds, Num_Rings, Num_AromaticRings and Molecular_FractionalPolarSurfaceArea. these descriptors were not pre-scaled. The variances of PC1, PC2 and PC3 for UNPD and drugs in **Figure 2** were 0.506,0.202,0.136 and 0.427,0.315,0.099, respectively.

### 4. Constructing of DTNe

We downloaded the experimental binding data of natural products from BindingDB [32] on Oct. 21, 2011. Molecular structures were compared according to InChIKey to identify natural products in BindingDB. Those binding data which target had definite UniProt entry were retained. NPs and experimental targets were connected in Cytoscape [33] to construct the drug-target network based on experimental data (DTNe). The network properties and node centralities were calculated by network plugin and CentiBin [34].

### 5. Constructing of DTNd

The target proteins of approved Drugs in DrugBank were marked out with “Targets”. There were 4152 target proteins and we used the crystal or NMR structures in RCSB Protein Data Bank (http://www.rcsb.org/pdb/home/home.do) to screen potential lead compounds. The protein-ligand complex structures of target proteins of approved drugs in DrugBank were download and hetero atoms were removed and then hydrogen atoms were added by using Discovery Studio. The original ligands in the complex structures were used as reference compounds to judge the affinity of NPs to corresponding targets. For each target protein, the binding site was defined as a 40×40×40 Å cube centered on the occupied space of the original ligand with a spacing of 0.375 Å between the grid points. Docking was performed by autodock4.01 in DOVIS 2.0 [35] and parameters were listed in **File S1.** The procedure of constructing of drug-target network based on docking data (DTNd) was the same with that of constructing of DTNe.

## Results and Discussion

### 1. Statistics of Molecular Properties of Natural Products and Comparison between Natural Products and FDA-approved Drugs

Some important molecular descriptors of natural products in UNPD and FDA-approved drugs in DrugBank [36] were listed in **Table 2** **.** Typically, statistical means and standard deviations of natural products were larger than those of FDA-approved drugs. Consequently, these complex and diverse chemical structures of natural products would provide more polypharmacology by interacting with multiple target proteins [6].

Lipinski’s “rule of five” [37] which was derived from the statistics of oral drugs was often used in first screening. Although wemi-empirical rules are not necessarily valid [38], Lipinski’s “rule of five” can be used to help find drug-like molecules from large componds library. The drug-like properties basically contain four aspects which have their own limits: molecular weight should be less than 500 Da, hydrogen bond acceptors (HBA) should be less than 10, hydrogen bond donors (HBD) should be less than 5, partition coefficient AlogP should be less than five. Recently, Leeson emphasized a point that Lipinski's rule of five would mislead drug discovery because some effective drugs did not meet all four cut-off criteria [39]. We checked the satisfied conditions for “rule of five” of all natural products in UNPD and found that only 102605 (52.0%) out of 197201 natural products met “rule of five” (**Table 3**). However, 141628 (71.8%) natural products met at least three cut-off criteria. Meanwhile, 1065 drugs, 77.17% of the total (1380), obey the “rule of five”. **Table 3** shows the count of the molecules obeying all the four limitations or three of them which shows a small fluctuation between different cut-off criteria. This is reasonable for that if molecular weight is bigger, the hydrogen bond acceptors or donors may become more at the same time. And AlogP has definitely the same relationship with these properties.

**Table 3:** Statistics of satisfied conditions for “rule of five” of natural products in UNPD and approved small drugs in DrugBank.

Rule of five	UNPD (total 197201)	DrugBank (total 1380)
all satisfied	102605	1065
except mw	113008	1074
except acceptors	103701	1074
except donors	106105	1081
except AlogP	126629	1150

UNPD contained a fair number of molecules only published in Chinese publications or even some of them have not be published till now. We compared UNPD molecules with FDA-approved drugs in several properties which have been mentioned before in “rule of five”. The histograms (**Figure 1**) of each descriptor of molecules in UNPD (197201 molecules) and Drugs (1380 molecules) showed that a vast majority of properties in two groups had a very similar distribution (both are non-normal distributions), which indicated that natural products can be a drug-like molecule resource for drug development. Considering our huge size of UNPD, this result will be more persuasive. From the histogram of molecule weight, drugs tended to be smaller than natural products. Most drugs were in the [250,300] interval while natural products were in the [300,350] interval. And natural products had less chiral centers. In the interval of less than 5 in histogram of ALogP, the distributions of NPs and drugs were quite similar. However, we still found that NPs had large ALogP which indicated that they would not dissolve in water easily. Provided that the solubility has large impact to therapeutic effectiveness, the distribution of ALogP may provide useful information.

### 2. Drug-like Space and Lead Compounds Discovery from Natural Products

The widely used concept of drug-like chemical space was important for drug discovery [23], [39]–[44]. Rosen and colleagues analyzed the chemical space occupancy of natural products and found that natural products exhibited similar activity to drugs with their neighborhood [24]. By using FDA-approved drugs as a reference in chemical space, we can screen potential lead compounds from large chemical libraries [41]. Drugs tended to have more aromatic or heterocyclic and less chiral centers, which was in agreement with the data in a recent study [45]. The median and mean of F-Chirality (number of chiral carbon atoms divided by total carbon count) in DrugBank and Natural products are 0.44, 0.38 and 0.45, 0.41, respectively. It shows that drugs had larger proportion of chiral centers than that of natural products. However, natural products had more carbons and so the total counts of chiral carbon are larger than that of drugs. Other properties were smaller than those of natural products, respectively. To get a better understanding of two groups of molecules, principal component analysis was employed to give visual illustration in chemical space. The 3D plot in **Figure 2** offered us an opportunity to compare the distribution between the NPs and drugs easily. The wide distribution in chemical space indicated that there would be vast property diversity in NPs. The large overlap in chemical space showed that natural products could be a large source for drug discovery.

### 3. Biological Activity of Natural Products

**Figure 3:**
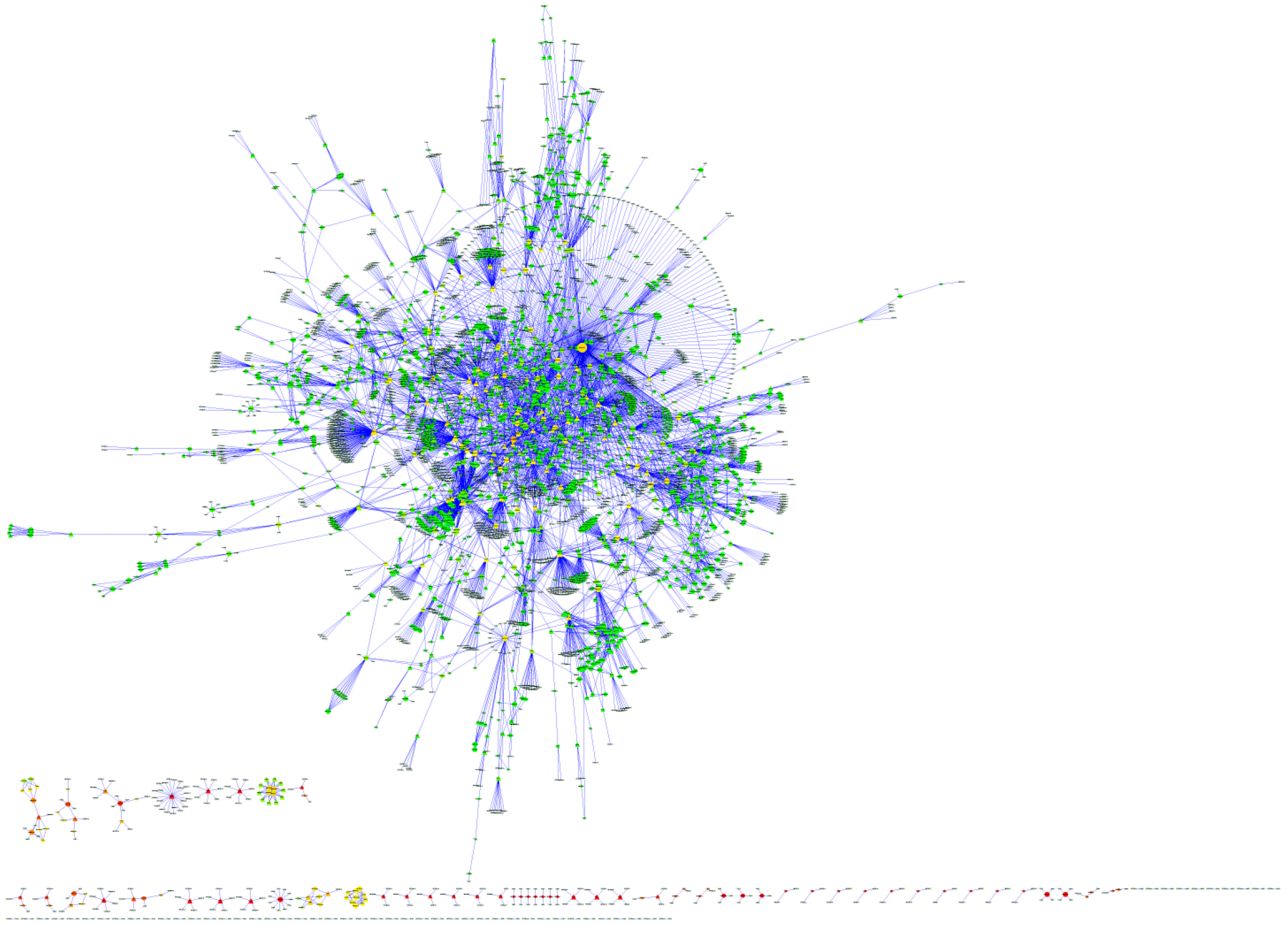
Drug-target network of natural products and their experimental targets (DTNe). The size of each node is proportional to its degree. The nodes are colored according to their shortest-path betweenness in the network. Circles and triangles correspond to small compounds (natural products or drugs) and target proteins, respectively.

Natural products have many biological activities and they can interact with multiple cellular targets since they are created by nature [6]. Presently, more than 17,000 records of such interactions have been reported according to BindingDB [32] and ChEMBL [46]. We extracted these interaction information (**Tables S1**) and constructed a drug-target network (DTNe) by connecting the natural products and their experimental targets (**Figure 3**).

Degree and betweenness centrality were two primary parameters to evaluate the importance of nodes in a network. Degree was defined as the number of neighbors of a node in a undirected graph. Betweenness reflected the important role nodes would play in information transmission in the network. Nodes with the highest local connectivity and the highest global centrality measured by degree and betweenness centrality were defined as hubs and bottlenecks, respectively [47]. Such nodes would be highly influential in the whole network.

DTNe was a typical scale-free network (degree distribution P(x) = 180.77*×∧(−1.125), r = 0.84), like most biological networks. This would be very important for network robustness and information transmission. Most natural products had only one or two experimental targets, and the average was 2.66. However, there were several natural compounds who had many targets, such as UNPD68000 (298 targets) and UNPD49205 (82 targets). UNPD68000 (staurosporine, STS) was a natural product isolated from the bacterium Streptomyces [48]. The main biological activity of STS was the inhibition of protein kinases by occupying the ATP-binding site of the target, with a high affinity and low selectivity. Staurosporine was was also the precursor of midostaurin which was a novel potent kinase inhibitor [49]. Right now, several staurosporine cognates are in advanced clinical trials for anticancer [50].

UNPD49205 (quercetin) was a flavonoid widely distributed in plants. As an antioxidant, it was similar to many other phenolic heterocyclic compounds. Quercetin has been effective against a wide variety of diseases, such as viral disease [51], [52], inflammations [53], and even cancer [54]. Moreover, several cellular models as well as animal models showed that the quercetin can also exert a direct effect in blocking the growth of tumor cells in different phases [55].

STS and quercetin had not only large degree but also high betweenness centrality. However, some natural products had low degree but high betweenness centrality in DTNe. UNPD152676 (genistein) was a well-known isoflavone in several plants. There were many biological functions of genistein reported to date, such as antioxidation and inhibition of epidermal growth factor receptor [55]. It was also reported that it can be potentially used to inhibit the growth of tumor cells [56].

Natural products have extensive biological activities and so can be used as a chemical library for drug discovery. However, there was lack of adequate information of the interactions between natural products and cellular targets. Fortunately, with the increasing development of computer technology, high throughput virtual screening gives us such ability to generate sufficient data. As a result, molecular docking by AutoDock4 [57] was adopted to simulate the interactions between natural products and cellular targets.

### 4. Network Pharmacology

Network pharmacology was proposed by Hopkins [58], [59] in 2007 and it could take advantage of network analysis methods to explore the pharmaceutical action of molecules in the context of biological networks. By analyzing the network properties or exploring the influence of compounds to the biological networks, it help us to understand the action mechanism and to evaluate the drug efficacy [14], [60]. Now network pharmacology is regarded as the next paradigm in drug discovery [59].

Because there were only 1.8% natural products which biological activities have been reported, we have an urgent need to obtain a large quantity of binding data between natural products and target proteins. By using Autodock4, all natural products were docked to 332 target proteins (all have protein-ligand complex structures in RCSB protein data bank) of FDA-approved drugs and screened according to docking score.

**Figure 4:**
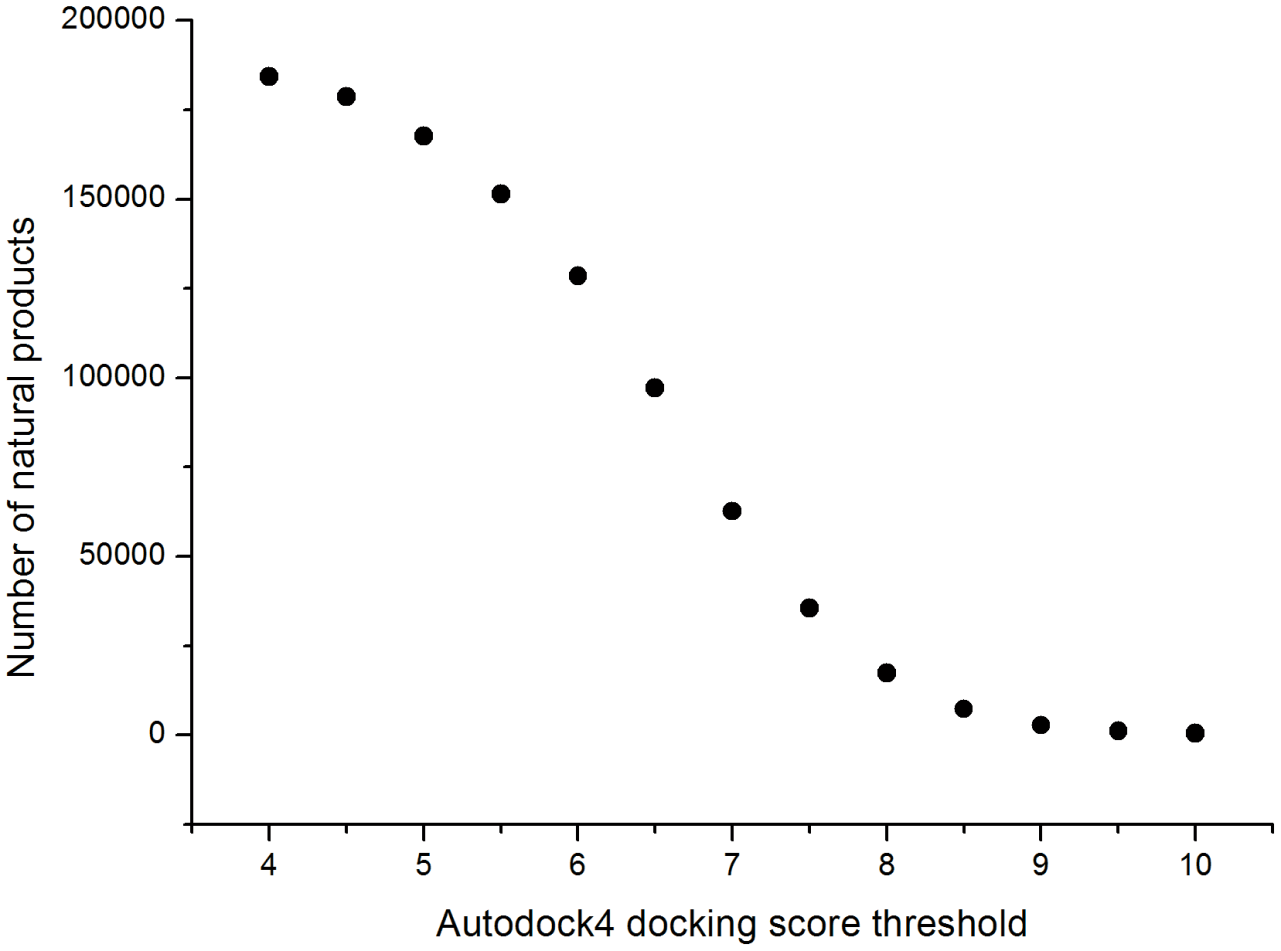
Distribution of docking score of natural products.

**Figure 5:**
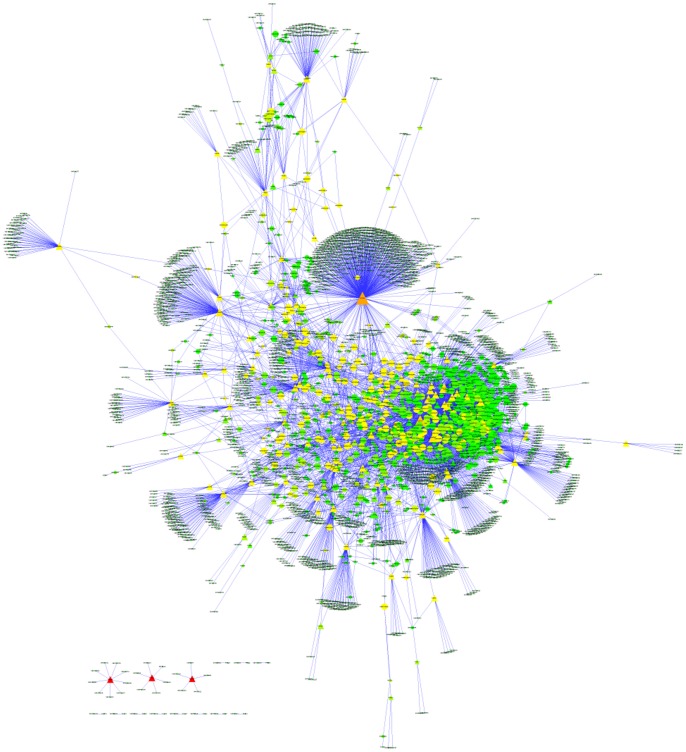
Drug-target network of natural products and their computational targets. Representations of the symbols are the same to Figure 3.

UNPD contained more than 65 millions of docked conformations of natural products and FDA-approved drugs. Although the potential binding of natural products in cavities that may be different from the binding site of drugs, most proteins had limited binding sites. In most cases, the binding sites of natural products and drugs were essentially the same.Generally, the hit rate of virtual screening is about 35% [61]. In this work, the number of natural products which docking score was higher than 7 and higher than the score of original ligand of complex structure of the target protein was 62918, accounting for 32% of total compound (**Figure 4**). Consequently, it would be an criterion to predict whether a natural product has certain kind of biological activity. In order to promote the accuracy of predicted results and lower the complexity of data handling, we set the threshold as that the docking score was higher than 9 and higher than the score of original ligand of complex structure of the target protein. Then we constructed drug-target network (DTNd, **Figure 5**). Typically, a natural product was linked to a target protein if the docking score exceeded the threshold (**Table S2**).

Natural products targeted at an average of 2.14 target proteins in DTNd and each target protein contained an average of 25 hits (natural products). Meanwhile, the two values of DTNe were 2.66 and 5.35 (**Table 4**), respectively. It would mean that most natural products have not conducted experimental test of biological activity. DTNd was comprised of 15 subgraphs. The giant component (the largest connected subnetwork) contained 2810 natural products and 228 target proteins, that is, accounting for 98.6% of all nodes. However, DTNe was comprised of 110 subgraphs and the giant component accounted for 90.1% of total nodes. Therefore, present studies on biological activities of natural products were far from systematic and molecular docking in a large-scale would be an effective supplement.

**Table 4:** General characteristics of three drug-target networks.

DTN	No. of compounds	No. of targets	<node degree>	<shortest path>	network density
DTNd	2884	243	3.96	4.30	0.0013
DTNe	2840	1413	3.56	5.95	0.0008
DTN*	1279	1328	3.68	7.16	0.0014

* Drug-target network of FDA-approved drugs and their pharmacological targets in DrugBank.

Most nodes in DTNd had high degree centrality. Especially, UNPD43323, UNPD194973, UNPD107682 and UNPD141622 (**Table 5**) had more than forty targets. These natural products would be noteworthy because polypharmacology is greatly enriched for high-degree compounds. UNPD43323, UNPD194973, UNPD129237, UNPD162694 and UNPD10433 had highest betweenness centrality, and the first two were also those compounds with largest degree.

**Table 5:** Most potential natural products for lead discovery.

UNPD ID	chemical name	CAS	Degree	Betweenness
UNPD43323	Ormojine	14710-67-9	90	0.072
UNPD194973	Ormosinin	NOT Available	63	0.035
UNPD107682	vatamidine	129741-48-6	46	0.014
UNPD141622	Vatamine	129741-49-7	40	0.020
UNPD61603	strychnohexamine	442123-70-8	35	0.017
UNPD38223	caledonine	235099-24-8	31	0.009
UNPD21224	Lycopodium Base B	54352-31-7	28	0.012
UNPD5255	Vatine	129741-50-0	28	0.005
UNPD41999	Lycopodium Base A	54352-30-6	26	0.005
UNPD2675	Seldomycin 5	56276-26-7	25	0.004

### 5. Predicted Diseases for Natural Products

**Figure 6:**
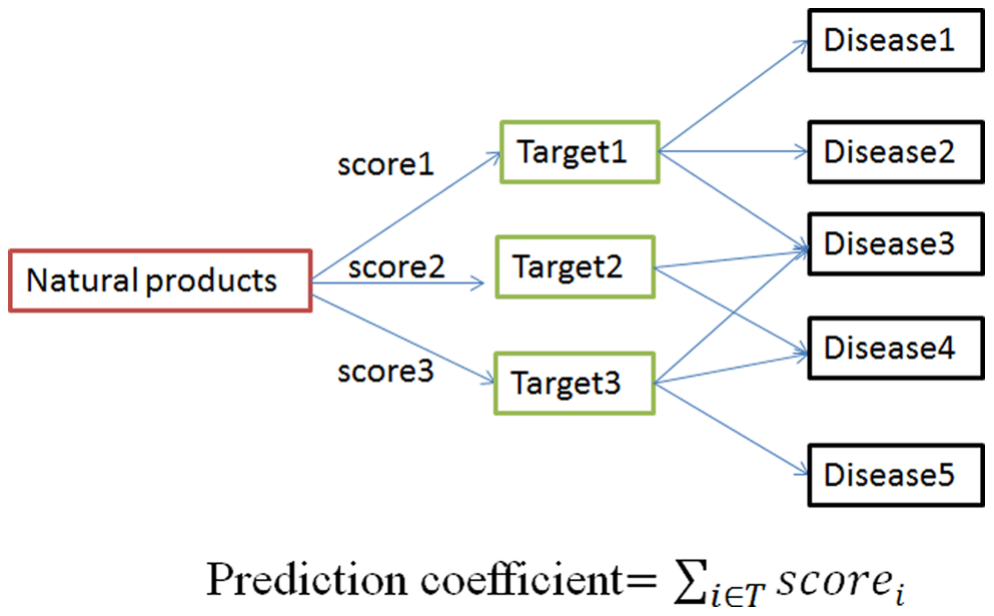
Prediction model of indications for natural products.

Natural products have been used to treat diseases for thousands of years. However, the molecular mechanism was rarely elucidated clearly. Here, we predicted the potential indications for natural products based on DTNd. Typically, natural products, especially high-degree compounds, would interact with several target proteins and target protein would concern a lot of diseases. After extracting the target-related diseases from Therapeutic Targets Database [62], we constructed a docking score-weighted prediction model (**Figure 6**) to predict the possibility of a natural product to treat some diseases (**Table 6** and **Table S3**). Typically, UNPD194973 and UNPD43323 would have very large latent capacity as drugs for bacterial infections and several cancers.

**Table 6:** Predicted indications for natural products.

Naturalproducts	Prediction coefficient	Indications
UNPD194973	58.12	Bacterial infections
UNPD43323	55.26	Prostate cancer
UNPD43323	51.60	Asthma
UNPD43323	50.08	Cancer, unspecific
UNPD107682	49.74	Bacterial infections
UNPD194973	47.89	Prostate cancer
UNPD112143	47.62	Prostate cancer
UNPD194973	44.34	Asthma
UNPD43323	43.37	Bacterial infections
UNPD107682	41.47	Prostate cancer
UNPD141622	39.76	Bacterial infections
UNPD141622	39.62	Prostate cancer
UNPD141622	39.00	Lung Cancer
UNPD141622	39.00	Osteoarthritis
UNPD194973	38.99	Cancer, unspecific
UNPD107682	38.24	Asthma
UNPD112143	37.97	Non-small Cell Lung Cancer
UNPD43323	35.82	Diabetes mellitus
UNPD43323	34.29	Non-small Cell Lung Cancer
UNPD43323	33.89	Brain Cancer

## Conclusions

Natural products have vast chemical diversity, not only structural diversity but also various biological activity, so as to guarantee the opportunities to find different kinds of lead compounds for different diseases. We find that NPs and FDA-approved drugs share a lot of space in chemical space. Moreover, NPs have a large quantity of lead-like molecules, which could be used as scaffolds to expand the chemical library.

Notwithstanding the recent advances in omics, the data collection of NPs is largely incomplete. First of all, the inventory of NPs remains incomplete and new chemical structures are being discovered [7]. Secondly, researchers explored only a small part of biological functions of NPs. Thirdly, there were mistakes and errors in existing data. Many chemical structures of NPs are questionable. Data of biological activity obtained from different laboratories for one compounds would vary greatly. While no adequate data is available, a good and useful complement is virtual screening results. Last but not least, more research methods both experimental and computational to afford more overall and more accurate data are needed urgently. We are extending the computational targets to all proteins if it has protein-ligand complex structure.

Presently, most studies on network pharmacology are based on static networks. However, biological networks is always changing. Recently, Hoeng and colleagues proposed that using of network analysis to prediction the efficacy or toxicity for chronic diseases by estimating the perturbation of biological networks would be particularly useful [60].

## Supporting Information

Table S1 **Lists experimental interaction between natural products and target proteins.**
(XLSX)

Table S2 **Lists computational interaction between natural products and target proteins.**
(XLSX)

Table S3 **Lists the prediction of indications for natural products.**
(XLSX)

File S1 **Lists the parameters used in the virtual screening by autodock4.0.**
(DOCX)
