# Supplementary material for: Use of Natural Products as Chemical Library for Drug Discovery and Network Pharmacology
Source: PLoS One. 2013 Apr 25;8(4):e62839. doi: 10.1371/journal.pone.0062839 (PMC3636197; doi:10.1371/journal.pone.0062839)
Supplement: File S1 — Lists the parameters used in the virtual screening by autodock4.0. (DOCX) [file pone.0062839.s004.docx]

Use of natural products as chemical library for drug discovery and network pharmacology

Jiangyong Gu^†^, Yuanshen Gui^†,‡^, Lirong Chen*^,†^, Gu Yuan^†^, Hui-Zhe Lu^†,‡^, Xiaojie Xu^*,†,^

^†^Beijing National Laboratory for Molecular Sciences, State Key Lab of Rare Earth Material Chemistry and Applications, College of Chemistry and Molecular Engineering, Peking University, Beijing 100871, P. R. China.

^‡^Institute of Science and Technology, China Agricultural University, Beijing 100193, PR China.

The supporting information has one file and three tables: **File S1** lists the parameters used in the virtual screening by autodock4.0. **Table S1** and **Table S2** are the lists of experimental and computational interaction between natural products and target proteins, respectively. **Table S3** lists the prediction of indications for natural products.

**File S1: the parameters used in the virtual screening by autodock4**

**DPF_predefined4:**

outlev 1 # diagnostic output level

ligand_types H HD HS C A N NA NS OA OS F P SA S Cl CL Br BR I # ligand atom types

fld .maps.fld # grid_data_file

map .H.map # atom-specific affinity map

map .HD.map # atom-specific affinity map

map .HS.map # atom-specific affinity map

map .C.map # atom-specific affinity map

map .A.map # atom-specific affinity map

map .N.map # atom-specific affinity map

map .NA.map # atom-specific affinity map

map .NS.map # atom-specific affinity map

map .OA.map # atom-specific affinity map

map .OS.map # atom-specific affinity map

map .F.map # atom-specific affinity map

map .P.map # atom-specific affinity map

map .SA.map # atom-specific affinity map

map .S.map # atom-specific affinity map

map .Cl.map # atom-specific affinity map

map .CL.map # atom-specific affinity map

map .Br.map # atom-specific affinity map

map .BR.map # atom-specific affinity map

map .I.map # atom-specific affinity map

elecmap .e.map # electrostatics map

desolvmap .d.map # desolvation map

tstep 2.0 # translation step/A

qstep 50.0 # quaternion step/deg

dstep 50.0 # torsion step/deg

torsdof 5 0.274000 # torsional degrees of freedom and coefficient

rmstol 2.0 # cluster_tolerance/A

extnrg 1000.0 # external grid energy

e0max 0.0 10000 # max initial energy; max number of retries

ga_pop_size 150 # number of individuals in population

ga_num_evals 1000000 # maximum number of energy evaluations

ga_num_generations 27000 # maximum number of generations

ga_elitism 1 # number of top individuals to survive to next generation

ga_mutation_rate 0.02 # rate of gene mutation

ga_crossover_rate 0.8 # rate of crossover

ga_window_size 10 #

ga_cauchy_alpha 0.0 # Alpha parameter of Cauchy distribution

ga_cauchy_beta 1.0 # Beta parameter Cauchy distribution

set_ga # set the above parameters for GA or LGA

sw_max_its 300 # iterations of Solis & Wets local search

sw_max_succ 4 # consecutive successes before changing rho

sw_max_fail 4 # consecutive failures before changing rho

sw_rho 1.0 # size of local search space to sample

sw_lb_rho 0.01 # lower bound on rho

ls_search_freq 0.06 # probability of performing local search on individual

**DPF_repeated4:**

seed 1 1 # for random number generator

move $LIGAND_PDBQ_NAME$ # small molecule file

tran0 random # initial coordinates/A or "random"

quat0 random # initial quaternion or "random"

dihe0 random # initial torsions

set_ga # set the above parameters for GA or LGA

set_sw1 # set the above pseudo-Solis & Wets parameters

ga_run 1 # do this many GA or LGA runs

#analysis # do cluster analysis on results

**GPF_predefined4:**

npts GRID_DIMENSION_NPT # num.grid points in xyz

gridfld $protein_name$.maps.fld # grid_data_file

spacing GRID_SPACING # spacing(A)

receptor_types H HD HS C A N NA NS OA OS F Mg MG P SA S Cl CL Ca CA Mn MN Fe FE Zn ZN Br BR I # receptor atom types

ligand_types H HD HS C A N NA NS OA OS F P SA S Cl CL Br BR I # ligand atom types

receptor PROTEIN_PDBQT # macromolecule

gridcenter GRID_CENTER # xyz-coordinates or auto

smooth 0.5 # store minimum energy w/in rad(A)

map $protein_name$.H.map # atom-specific affinity map

map $protein_name$.HD.map # atom-specific affinity map

map $protein_name$.HS.map # atom-specific affinity map

map $protein_name$.C.map # atom-specific affinity map

map $protein_name$.A.map # atom-specific affinity map

map $protein_name$.N.map # atom-specific affinity map

map $protein_name$.NA.map # atom-specific affinity map

map $protein_name$.NS.map # atom-specific affinity map

map $protein_name$.OA.map # atom-specific affinity map

map $protein_name$.OS.map # atom-specific affinity map

map $protein_name$.F.map # atom-specific affinity map

map $protein_name$.P.map # atom-specific affinity map

map $protein_name$.SA.map # atom-specific affinity map

map $protein_name$.S.map # atom-specific affinity map

map $protein_name$.Cl.map # atom-specific affinity map

map $protein_name$.CL.map # atom-specific affinity map

map $protein_name$.Br.map # atom-specific affinity map

map $protein_name$.BR.map # atom-specific affinity map

map $protein_name$.I.map # atom-specific affinity map

elecmap $protein_name$.e.map # electrostatic potential map

dsolvmap $protein_name$.d.map # desolvation potential map

dielectric -0.1465 # <0, AD4 distance-dep.diel;>0, constant

**dovis_input:**

TOTAL_CPU 8

LOAD_SIZE 1

LIGAND_LIST ligand_list

PARALLEL_MODE THREADING # THREADING, SSH or LSF

LOCK_SLEEP_TIME 1

LOCK_RETRIES 100

KEEP_WORK_DIR NO

INITIAL_SLEEP NO

#WORKING_DIR /usr/tmp/xjiang

#RUNTIME_LIMIT 5:00 # hour:min

ALARM_OFFSET 15 # min

TIMER_FACTOR 1.5

NUM_SAVED_LIG ALL

SCORE_ORDER DOWN # DOWN or UP

AUTODOCK_MODE POLAR_H_AA # ALL_ATOM, POLAR_H, POLAR_H_AA

SCORE_LABEL 'AD4_score'

CLUSTER_NUM 3

CLUSTER_RMS 1.5

#SCORING_COMMAND run_xscore.pl -c 'xscore -score'

#PARAMETER_FILES box.sd
